# Associations Between Mobile Health Technology use and Self-rated Quality of Life: A Cross-sectional Study on Older Adults with Cognitive Impairment

**DOI:** 10.1177/23337214211018924

**Published:** 2021-05-25

**Authors:** Line Christiansen, Johan Sanmartin Berglund, Peter Anderberg, Selim Cellek, Jufen Zhang, Evi Lemmens, Maite Garolera, Fermin Mayoral-Cleries, Lisa Skär

**Affiliations:** 1 Blekinge Institute of Technology, Karlskrona, Sweden; 2 University of Skövde, Sweden; 3 Anglia Ruskin University, Bishop Hall Lane, Chelmsford, UK; 4 University Colleges Leuven-Limburg, Genk, Belgium; 5 Brain, Cognition and Behavior—Clinical Research, Consorci Sanitari de Terrassa, Barcelona, Spain; 6 Regional University Hospital of Málaga, Spain

**Keywords:** aging, cognitive impairment, gerontechnology, mobile health, quality of life

## Abstract

**Background:** Quality of life (QoL) is affected even at early stages in older adults with cognitive impairment. The use of mobile health (mHealth) technology can offer support in daily life and improve the physical and mental health of older adults. However, a clarification of how mHealth technology can be used to support the QoL of older adults with cognitive impairment is needed. **Objective:** To investigate factors affecting mHealth technology use in relation to self-rated QoL among older adults with cognitive impairment. **Methods:** A cross-sectional research design was used to analyse mHealth technology use and QoL in 1,082 older participants. Baseline data were used from a multi-centered randomized controlled trial including QoL, measured by the Quality of Life in Alzheimer’s Disease (QoL-AD) Scale, as the outcome variable. Data were analyzed using logistic regression models. **Results:** Having moderately or high technical skills in using mHealth technology and using the internet via mHealth technology on a daily or weekly basis was associated with good to excellent QoL in older adults with cognitive impairment. **Conclusions:** The variation in technical skills and internet use among the participants can be interpreted as an obstacle for mHealth technology to support QoL.

## Introduction

The movement toward mobile health (mHealth) technology to meet the needs of an aging population is widely discussed as beneficial (Changizi & Kaveh, 2017; Sohaib Aslam et al., 2020). However, there are still concerns that need addressing before mHealth can meet its potential, which include examination of the ways in which digital health technologies can support health and quality of life (QoL) in older adults (Lupton, 2018; Marston et al., 2017). Concerning older adults with cognitive impairment, deterioration in memory, and other cognitive domains increases with age and affects QoL even at early stages (Bárrios et al., 2013; Winblad et al., 2016).

Longitudinal studies on the progression of cognitive impairment show that people with mild cognitive impairment are at higher risk of developing dementia, a multifactorial disorder known to be burdensome to older persons and their networks and a major cause of functional dependence, institutionalization, poor QoL, and mortality in older adults (Johansson et al., 2015; Prince et al., 2015; Qiu et al., 2013). There is an increasing number of studies suggesting that the adoption of mHealth technology can offer support in daily activities, relationships, memory, leisure activities, health, and safety; thus, it may improve the physical and mental health of older adults (Koo & Vizer, 2019; Rathbone & Prescott, 2017). However, according to previously published research, using the same source of information, the technology literacy level related to the use of mHealth technology has been shown to vary significantly among older adults with cognitive impairment, and there is a gap between the perceived potential and real use of these technologies (Christiansen et al., 2020; Guzman-Parra et al., 2020). In addition, the evidence for improving health and QoL with the use of mHealth technology among this study population is of limited quality; there is a lack of or inconsistency in data on health outcomes used for the evaluation of studies, little to no emphasis on user-centered design, study populations that are too small and so on (Bateman et al., 2017). What is obvious from the studies reported in the literature is that the relationship between mHealth technology and QoL has not yet been clarified. Determining this relationship contributes to bridging the knowledge gap of ways in which mHealth technology can be used to support QoL in older adults with cognitive impairment. Hence, the purpose of this study was to investigate factors affecting mHealth technology use in relation to self-rated QoL among older adults with cognitive impairment.

## Methods

### Design and Setting

A cross-sectional research design was used to investigate mHealth technology use and QoL among older adults with cognitive impairment. The present study used baseline data, collected between October 2017 and February 2019, from a multi-center randomized controlled trial—the Support, Monitoring and Reminder Technology for Mild Dementia project (SMART4MD; www.smart4md.eu; Anderberg et al., 2019). The trial was carried out in four clinical centers located as follows: one in Belgium, two in Spain, and one in Sweden. The objective of the trial was to investigate the effects of a customized mHealth application on the QoL of older adults with mild dementia or mild cognitive impairment and their caregivers. The application has been adapted specifically for this study population through a structured process involving the participation of primary users (adults with cognitive impairment) and informal caregivers. The protocol is registered at ClinicalTrials.gov (NCT03325699).

### Participants

In total, 1,082 older participants were selected from the SMART4MD trial to be included in this study. The selection was based on the same inclusion criteria as used in the trial, where the participants needed to be aged 55 or above, have an informal caregiver, and have experienced difficulties in recall for the last 6 months. The participant also needed to score between 20 and 28 points on the Mini–Mental State Examination (MMSE) to be included. The MMSE contains questions regarding memory, learning, orientation, and so on; the possible score is 0 to 30 points, where a score of 26 points or less indicates cognitive difficulties (Folstein et al., 1975). In this study, a cut-off of 28 points was used based on findings that a cut-off score of 27 or 28 is appropriate to use in larger evaluations because adults in this context are at a greater risk of being diagnosed with dementia (O’Bryant et al., 2008). The median MMSE score for participants was 26 (interquartile range [IQR] = 24–28) points, and 28.70% (*n* = 300) had received the formal diagnosis of dementia. Participants who scored 11 or above on the Geriatric Depression Scale or had a life expectancy of 3 years or less were excluded. This study sample has been included in a previous study (Guzman-Parra et al., 2020).

### Measures

#### Outcome variable

To measure QoL as the outcome variable in this study, the Quality of Life in Alzheimer’s Disease (QoL-AD) Scale was used (Logsdon et al., 1999). This is a disease-specific questionnaire, measuring the current QoL in individuals with cognitive impairment/dementia based on 13 items with a 4-point Likert scale, where the highest score responds to excellent QoL and the lowest score responds to poor QoL. The QoL-AD is administered in an interview format where specific items have been selected to reflect Lawton’s four domains of QoL in older adults. The internal reliability of the questionnaire was established in patients with AD and their caregivers’ it was later found to be reliable and valid for individuals with MMSE scores >10 (Logsdon et al., 2002). In this study, the Cronbach’s alpha for the QoL-AD index was calculated to be 0.886, indicating good reliability. To establish the limit for poor to fair and good to excellent QoL among the study population, a cut-off on the 25th percentile (equal to a score of 32) in the QoL-AD index was used.

#### Variables

Sociodemographic characteristics such as age, sex, education level, and living arrangement was included to control for the main associations and whether the sample reflected the general population of this study or not. Cognitive status included the presence or absence of a formal diagnosis of dementia. To study mHealth technology use in relation to QoL, variables on access to the internet, self-assessed technical skills, frequency of usage, and attitude toward mHealth technology were included (Anderberg et al., 2019). These variables were used to assess the participants’ perception of using mHealth technology and the inclusion were based on previous findings from a qualitative study and a feasibility study using the same sample (Christiansen et al., 2020; Quintana et al., 2020).

### Statistical Analysis

All statistical analyses were performed using the Statistical Package for Social Science (SPSS), version 26.0 (IBM Corp., Armonk, NY). An initial descriptive analysis was conducted on the self-rated QoL in the QoL-AD, where the median value and the interquartile range (IQR) were calculated for the participants’ response scores. When analyzing all the variables, the Chi-Square test and Mann–Whitney *U*-test were used in the comparison of poor to fair and good to excellent QoL. These results are presented as relative frequency (%) and absolute frequency (*N)*. To analyse the association between mHealth technology use and self-rated QoL, univariate analysis (i.e., correlations with Spearman’s rho [r_s_] and binary logistic regression) and multivariate logistic regression models were performed. For model comparisons, the likelihood ratio (forward LR) was used in a stepwise selection based on the significance of the score statistic and on the probability. To determine how well the observed data corresponded to the predicted data in the models, the likelihood ratio test and goodness-of-fit test of Hosmer and Lemeshow (2013) was used. The results of the final multivariate logistic regression model are presented as odds ratios (ORs) with their 95% confidence intervals (CIs) and *p*-values for statistical significance (*p* < .05).

## Results

In this study, the proportions of gender and age were similar, where 53.10% (*N* = 575) were women, with a median age of 75 (IQR = 70–79) years, and 46.90% (*N* = 507) were men, with a median age of 75 (IQR = 71–79) years. In the study sample, the proportions of participants with good to excellent and poor to fair QoL, as assessed using the QoL-AD, were 76.60% (*N* = 796) and 26.40% (*N* = 286), respectively.

### Self-rated QoL based on Different QoL Aspects

The median QoL score assessed by the QoL-AD among the study sample was 36.00 (IQR = 32.00–40.00), indicating a good QoL. The median score was slightly higher in men (38.00, IQR = 34.00–41.00) than it was in women (36.00, IQR = 31.00–39.00). Most participants reported that they had a good relationship with their spouse (3.00, IQR = 3.00–4.00), family members (3.00, IQR = 3.00–4.00), and friends (3.00, IQR = 3.00–3.00) and felt they had a good living situation (3.00, IQR = 3.00–4.00). The lowest median value of QoL was observed for the participants’ self-rated memory, where most reported having either poor or fair memory (2.00, IQR = 1.00–3.00).

### Relationship Between mHealth Technology use and QoL

As shown in Table 1, the greatest proportions of participants who reported poor to fair QoL had the following characteristics: female sex (64.40%, *N* = 184), age of 65–74 years (40.60%, *N* = 116), completion of elementary school (72.90%, *N* = 207) and previous diagnosis of dementia (35.80%, *N* = 98). Among those who reported good to excellent QoL, higher responses were observed in terms of higher education level (22.80%, *N* = 181), technical skills in using mHealth technology (26.10%, *N* = 208) and frequency of using the internet with mHealth technology (38.20%, *N* = 304). As a coherent perception, most participants had a positive attitude toward using mHealth technology for memory support (75.80%, *N* = 820).

**Table 1.** Distribution of Variables by Self-Rated QoL among Older Adults with Cognitive Impairment (*N* = 1,082).

Variable	Good/excellent QoL	Poor/fair QoL	Total	*p*-Value^a,b^
	*N* (%)	*N* (%)	*N* (%)	
Gender				.00^a^
Male	405 (50.90)	102 (35.70)	507 (46.90)	
Female	391 (49.10)	184 (64.30)	575 (53.10)	
Age groups				.00^b^
55–64	49 (6.20)	49 (17.10)	98 (9.10)	
65–74	312 (39.20)	116 (40.60)	428 (39.60)	
75–84	371 (46.60)	108 (37.80)	479 (44.30)	
85+	64 (8.00)	13 (4.50)	77 (7.10)	
Education level (*n* = 1,077)				.00^b^
Elementary school	439 (55.40)	207 (72.90)	646 (60.00)	
Secondary school	173 (21.80)	51 (18.00)	224 (20.80)	
Higher education	181 (22.80)	26 (9.20)	207 (19.20)	
Living arrangement (*n* = 1,074)				.55^a^
Living with others	625 (79.10)	230 (81.00)	855 (79.60)	
Living alone	165 (20.90)	54 (19.00)	219 (20.40)	
Diagnosis of dementia (*n* = 1,045)				.00^a^
Yes	202 (26.20)	98 (35.80)	300 (28.70)	
No	569 (73.80)	176 (64.20)	745 (71.30)	
Access to internet (*n* = 1,013)				.04^a^
Yes	565 (74.20)	170 (67.50)	735 (72.60)	
No	196 (25.80)	82 (32.50)	278 (27.40)	
Frequency of using mHealth technology				.14^b^
Daily	448 (56.30)	144 (50.30)	592 (54.70)	
Weekly	65 (8.20)	31 (10.80)	96 (8.90)	
Rarely	21 (2.60)	8 (2.80)	29 (2.70)	
Never	262 (32.90)	103 (36.00)	365 (33.70)	
Frequency of using the internet with mHealth technology				.002^b^
Daily	304 (38.20)	76 (26.60)	380 (35.10)	
Weekly	71 (8.90)	23 (8.00)	94 (8.70)	
Rarely	41 (5.20)	28 (9.80)	69 (6.40)	
Never	380 (47.70)	159 (55.60)	539 (49.80)	
Technical skills in using mHealth technology				.00^b^
None	278 (34.90)	131 (45.80)	409 (37.80)	
Low	268 (33.70)	118 (41.30)	386 (35.70)	
Moderately	208 (26.10)	34 (11.90)	242 (22.40)	
High	42 (5.30)	3 (1.00)	45 (4.10)	
mHealth technology for memory support				.60^a^
Yes	155 (19.50)	51 (17.80)	206 (19.00)	
No	641 (80.50)	235 (82.20)	876 (81.00)	
App/software for memory support				.73^a^
Yes	79 (9.90)	26 (9.10)	105 (9.70)	
No	717 (90.10)	260 (90.90)	977 (90.30)	
Attitude toward mHealth technology for memory support				.02^a^
Positive	618 (77.60)	202 (70.60)	820 (75.80)	
Negative	178 (22.40)	84 (29.40)	262 (24.20)	

*Note.* Significance level *p* < .05.

a Pearson Chi-square.

b Mann**–**Whitney *U*-test.

In the logistic regression analysis, univariate analysis showed weak correlations with attitudes toward mHealth (*r_s_* = 0.07, *p* = .02), access to the internet (*r_s_* = 0.07, *p* = .04), frequency of using the internet with mHealth technology (*r_s_* = 0.11, *p* < .001) and technical skills in using mHealth technology (*r_s_* = 0.18, *p* < .001). In the multivariate analysis, two of the mHealth variables was found to be associated with QoL (Table 2). Those who reported having moderately or high technical skills in using mHealth technology had 127% (OR = 0.44) higher odds of having good to excellent QoL than those who reported having no or low technical skills. Further, those who reported using the internet daily or weekly with mHealth technology had 55% (OR = 0.65) higher odds of having good to excellent QoL than those who rarely or never used the internet.

**Table 2.** Multivariate Logistic Regression Analysis (Forward: LR). Impact of mHealth Technology Use on Self-Rated QoL (*N* = 1,077).

Coefficient OR (e^b^)		95% CI for OR	*p*-Value
Constant	5.04	—	0
Gender			
Male	Ref.		
Female	0.62	0.46**–**0.86	0
Age groups			
55–64	Ref.		
65–74	2.6	1.57–4.30	0
75–84	4.79	2.82–8.14	0
85+	6.51	2.85–14.85	0
Education level			
Elementary school	0.5	0.31–0.82	.01
Secondary school	0.74	0.42–1.31	.3
Higher education	Ref.		
Technical skills in using mHealth technology			
None/low	0.44	0.28–0.69	0
Moderately/high	Ref.		
Frequency of using internet with mHealth technology			
Daily/weekly	Ref.		
Rarely/never	0.65	0.44–0.94	.02
Test		χ^2^	***p*-Value**
Overall model evaluation			
Likelihood ratio test		103.93	0
Goodness-of-fit test			
Hosmer and Lemeshow		4.07	.77

*Note.* Cox and Snell *R*^2^ = 0.10. Nagelkerke’s *R*^2^ = 0.15. QoL = quality of life is dichotomized (good/excellent QoL = reference).

Overall, the multivariate logistic regression analysis resulted in a model that explains 15% of the variation in the incidence of having good to excellent QoL (Table 2). The rate of having good to excellent QoL was 60% (OR = 0.62) higher among men than women and increased with age (OR = 2.60–6.51). Those who had completed higher education had 99% (OR = 0.50) higher odds of having good to excellent QoL compared with those who completed elementary school.

## Discussion

This study aimed to investigate factors affecting mHealth technology use in relation to self-rated QoL among older adults with cognitive impairment. The results showed that the self-rated QoL among the study sample was generally perceived as good, but poorer QoL was reported in relation to the participants’ self-rated memory. As demonstrated in the analysis, those diagnosed with dementia had a poorer QoL. Despite this, cognitive status (i.e., diagnosis of dementia) was not included in the final model due to correlations with the mHealth variables. These results are in line with previous research that used the QoL-AD Scale, clarifying that QoL ratings by people with mild dementia are influenced by a variety of factors; thus, QoL cannot be determined by a single aspect, such as cognitive status (Woods et al., 2014). Since the measurement of QoL in this study is relevant both in relation to the participants’ condition and to the use of mHealth technology, the results must be considered from a multi-dimensional perspective, which includes micro (individual, subjective) and macro (societal, objective) perspectives on QoL (i.e., Bowling, 2017).

From a micro perspective, the results of this study showed that technical skills and frequency of using the internet was associated with the self-rated QoL, where moderately to high technical skills and daily or weekly internet use corresponded to the perception of having a good or excellent QoL among the older adults. Despite this, many considered that they had low technical skills in using mHealth technology, and almost half of the study sample reported that they never used the internet with mHealth technology. The proportion of daily internet use through mobile technology among individuals aged 16–74 years in Europe was 73% at the beginning of 2019, where Belgium, Spain, and Sweden recorded some of the highest proportions of mobile internet use (Eurostat, 2020). Previous research shows that self-assessed abilities and computer/internet skills among older adults are predictive of willingness to adopt technologies (Berkowsky et al., 2017). Studies also indicate that the use of the internet, especially online cognitive training programmes, may have a positive effect on cognitive functions in older adults (Berner et al., 2019; Klimova, 2016). However, using the internet via mHealth technology requires a range of cognitive and technical abilities, such as information-processing speed and working memory performance. In older adults with cognitive impairment, these cognitive abilities are usually reduced, which can negatively affect their use of and interaction with user interfaces (Kwon, 2017; Marston et al., 2017).

Although most older adults in this study had a positive attitude toward using mHealth technology to support memory, the variation in their technical skills, and internet usage indicates that the technology needs to be adapted. Admittedly, this result can be considered as a technology-related cohort effect, as older adults in the future will probably use different types of ICT to an increasing extent, including mHealth technology, to support functions in their daily lives, and maintain social networks. Regardless, a generational gap in capabilities will likely always remain (Damant & Knapp, 2015).

From a macro perspective, the results showed that the self-rated QoL was associated with gender, age, and level of education. The perception of having a good to excellent QoL was significantly higher among males, those with a higher age and those who had completed higher education. The gender differences in QoL among the study sample are probably linked to the level of education. For many years, men have had a higher level of education than women, which is mirrored in this study sample. In addition, higher levels of education are associated with better cognitive performance (Shuba & Prakash, 2017). Regarding gender differences in technology use, research shows that men are more likely to use and access various types of information and communication technology (ICT) than women are (Kim et al., 2017). Research also shows that internet use in old age predicts a smaller cognitive decline only in men (Ihle et al., 2020). Nevertheless, research on the gender differences in mHealth technology use among older adults with cognitive impairment is still limited.

The results of a higher QoL with increased age may be explained by respondents’ living circumstances. Since most participants reported having a good living situation, where 79.60% lived with their spouse, children or someone else, they had social interaction; previous studies identified social interaction as important for the perception of having a good QoL among older adults with mild cognitive impairment (Beerens et al., 2016; Christiansen et al., 2020). In addition, a socially integrated lifestyle in old age, along with mental and physical activity, can have a beneficial effect on cognition that may protect against dementia (Fratiglioni et al., 2004). Overall, the findings from this study contributes with knowledge of importance to the design of mHealth technology for older adults with cognitive impairment by showing ways of using mHealth technology. For the use of mHealth technology to support QoL in older adults with cognitive impairment, future intervention research must be responsive to the needs and capabilities of these older adults.

## Limitations

Since this study used a cross-sectional design, in which observed data were collected at a specific point in time, a causal relationship between mHealth technology use and QoL cannot be established. However, the use of a cut-off point for assessing QoL revealed valuable information on the variation in QoL within the study sample at baseline, which is of importance for future research when evaluating longitudinal effects of mHealth technology on QoL in older adults with cognitive impairment.

Further, the inclusion of variables on mHealth technology use in this study were limited to some of the technology use factors for this population which may have affect the final outcome model. However, there may be other factors such as general health status and financial support affecting the relationship between self-rated QoL and mHealth technology use than those included in this study. Also, the use of a disease-specific instrument to measure the outcome among a study sample in the intermediate stage between healthy aging with minor cognitive deficits and dementia can be regarded as a limitation on the validity of the results in this study. However, the inclusion of a rather large sample where almost 30% of the respondents had been diagnosed with dementia, and given that the respondents’ cognitive status is likely to deteriorate (Faria et al., 2018), the chosen instrument is considered valid for this study sample.

## Conclusions

Factors affecting mHealth technology use in relation to self-rated QoL among older adults with cognitive impairment involves sociodemographic factors, technical skills and internet use. Using mHealth technology can support QoL in older adults with cognitive impairment provided they have adequate technical skills to use the internet regularly. However, the variation in technical skills and internet use among these older adults seems to constitute an obstacle for mHealth technology to support QoL among this population. To facilitate the use and increase the adoption of mHealth technology among older adults with cognitive impairment, we recommend that future development and design of mHealth technologies be based on a participatory approach where older adults with cognitive impairment are integrated into the innovation process.

The findings from this study have implications for future evaluations of mHealth interventions aiming at supporting the QoL in older adults with cognitive impairment, mainly by demonstrating aspects that are important for mHealth technology use to contribute to an improved QoL, as well as by showing the variation of QoL within this population.
